# Supplementary material for: Nutritional approach based in self-compassion versus energy-restricted diet approach in body dissatisfaction and disordered eating in adult women: a protocol for randomized clinical trial
Source: PLoS One. 2025 Jun 2;20(5):e0324030. doi: 10.1371/journal.pone.0324030 (PMC12129175; doi:10.1371/journal.pone.0324030)
Supplement: Attachment — Submitted filename: Reviewer Attachments for Manuscript Number PONE-D-24-40704.docx [file pone.0324030.s002.docx]

Editor,

The study *“Nutritional approach based on self-compassion versus energy-restricted diet approach in body dissatisfaction and disordered eating in adult women: a protocol for a randomized clinical trial”* aimed to analyze the effects of a nutritional approach involving techniques that promote self-compassion compared to a traditional approach in women experiencing body image dissatisfaction, dietary restriction, and dysfunctional eating habits. I believe the article addresses an important and well-structured topic; however, I have a few questions:

**[Page 2 - Line 33]**
*Food restriction*
Would this be the most appropriate term? What about using *caloric restriction* or *caloric deficit* instead?

**[Page 2 - Line 44]**
“This will be the first study to compare these two approaches using body dissatisfaction and eating behavior as outcomes, not just body weight.”
In the discussion, it seems to justify the reasoning behind the authors' decision to conduct the study.

**[Page 3 - Line 60]
“**Although they show results in the short and medium term, such programs are ineffective in maintaining weight in the long term and can contribute to weight regain.”
It seems to imply that the programs are ineffective.

**[Page 4 - Line 78]
“**It is a practice that can be learned, accessible anytime, and may relieve suffering [23].”
The meditation practice requires time to master, yet here it gives the impression of being "easy."

**[Page 5 - Line 107]**
“Be excluded from the study if they have a diagnosis of depression, mood disorders (anxiety, bipolar disorder, borderline), eating disorders, or a history of suicidal ideation;”
Wouldn’t this sample be too restrictive? This exclusion criterion omits a broad group, often associated with body dissatisfaction.

**[Page 5 - Line 116]
“**Conversation via WhatsApp video call.”
Would it not be better to use Google Forms for registration to avoid needing to "disconnect" participants from the study? Calling each participant would be more time-consuming.

**[Page 7 - Line 144]
“**24-hour food recall (24HR).”
Using this questionnaire might lead to discrepancies in dietary intake reports, as one patient could respond on a Monday while another respond on a Wednesday.

**[Page 8 - Line 149]
“**The total energy value will depend on the participant's goal, with a calorie deficit of 300 kcal for those wishing to lose weight and a surplus of between 300–500 kcal for those wishing to gain weight.”
What criteria were used to determine the 300 kcal deficit? And for the 300–500 kcal surplus? How will this be implemented?

**[Page 10 - Line 177]
“**Before each meeting, participants will be notified via smartphone about the day, time, and place of the next meeting.”
Would not using pre-scheduled dates for meetings increase the dropout rate?

**[Page 10 - Line 191]
“**Translated into Brazilian.”
Would *“Translate to Portuguese”* be a more appropriate term?

**[Page 12 - Line 240]
“**Random Group Generator (<https://pt.rakko.tools/tools/59/>).”

Is it necessary to include the link? It could make the text appear "cluttered."

**[Page 14 - Line 267]
“**An interim analysis could be performed to present the study's preliminary results in scientific events. All authors could have access to interim results, but only the study coordinator (CGS) could decide to terminate the trial.”
Is it necessary to mention this?

Cordiality,
Gabriel Fassina Ladeia
